# GEAR 2.0 ‐ Care Transitions

**DOI:** 10.1002/alz.091842

**Published:** 2025-01-09

**Authors:** Manish N Shah

**Affiliations:** ^1^ University of Wisconsin‐Madison, Madison, WI, USA; University of Wisconsin School of Medicine and Public Health, Madison, WI USA

## Abstract

Older adults use the emergency department (ED) as an important source of acute illness care, making over 20 million visits annually. Persons living with dementia are twice as likely to use the ED and 1.5 times more likely to have an avoidable visit. When in the ED, they may not be able to give the clinicians a complete and accurate medical history, including information regarding their dementia. Further, they struggle with the environment and conditions. These challenges, along with other risks, put patients living with dementia at greater likelihood for poor outcomes.

Approximately 50 percent of patients with impaired cognition are discharged to their homes after receiving ED care, but approximately 40 percent of those discharged home will experience an adverse event (ED revisits, other hospitalizations, or death) in the 30 days following discharge—a rate significantly higher than for those without impaired cognition.

This session will review the interventions delivered to patients with impaired cognition to improve ED discharge transitions and the measures of quality ED discharge transitions that are important to ED patients with impaired cognition and their care partners.

